# Chronic obstructive pulmonary disease (COPD) literacy among traffic police in Kathmandu, Nepal: A cross-sectional study

**DOI:** 10.1097/MD.0000000000049972

**Published:** 2026-07-31

**Authors:** Sandhya Niroula, Kiran Paudel, Gaurab Bhattarai, Subash Wagle, Sangam Shah, Aashish Rana, Dikshya Parajuli, Rojina Dangol, Nikita Bhatta, Rhea Dhakal, Aayush Adhikari, Ashok Bhurtyal

**Affiliations:** a Central Department of Public Health, Institute of Medicine, Tribhuvan University, Kathmandu, Nepal; b Nepal Health Frontiers, Kathmandu, Nepal; c Institute of Medicine, Tribhuvan University, Maharajgunj, Nepal; d College of Medical Sciences, Bharatpur, Chitwan, Nepal.

**Keywords:** air pollution, chronic obstructive pulmonary disease (COPD), health literacy, Kathmandu, Nepal, traffic police

## Abstract

In Nepal, chronic obstructive pulmonary disease (COPD) is a growing concern, particularly among traffic police who are exposed to high levels of air pollution. Traffic police in Kathmandu are continuously exposed to air pollution and are at increased health risk. Therefore, we aimed to study COPD literacy among traffic police of Kathmandu Valley, Nepal. A descriptive cross-sectional study was conducted among 250 traffic police officers working at 11 sites in the Kathmandu Valley from July to August 2023. A 20- to 25-minute interview was conducted with randomly selected participants at the respective study location. Multivariable logistic regression was used to assess the factors associated with COPD literacy. The mean age of the participants was 30.5 (±6.6) years, and more than 3-quarters of the respondents had more than 5 years of experience. The study revealed that nearly half of the participants (48.8%) had limited knowledge of COPD. Traffic police who studied COPD in school (adjusted odds ratio [AOR] = 2.7, 95% confidence interval [CI] = 1.5–4.7), who heard about chronic respiratory diseases (AOR = 3.4, 95% CI = 1.7–6.6), and who never smoked (AOR = 1.9, 95% CI = 1.0–3.5) had higher odds of having a good level of knowledge of COPD. Unmarried participants had lower odds of having a good level of knowledge of COPD than the married participants (AOR = 0.4, 95% CI = 0.2–0.7). This study highlights inadequate COPD literacy among Kathmandu’s traffic police and emphasizes the need for targeted educational interventions. Integrating COPD education into school curricula, traffic police training programs, and public health initiatives is crucial, particularly for high-risk occupational groups such as traffic police. These findings underscore the role of health literacy in promoting preventive behaviors and reducing the burden of COPD among at-risk populations.

## 1. Introduction

Chronic obstructive pulmonary disease (COPD) refers to a group of diseases that cause airflow blockage and breathing related problems. It includes emphysema and chronic bronchitis.^[1]^ COPD is the third leading cause of death worldwide, causing 3.4 million deaths in 2023, and is the seventh leading cause of poor health worldwide (measured by disability-adjusted life years [DALYs]).^[2]^

The Global Burden of Disease 2023 report identifies COPD as one of the leading causes of death and disability in Nepal.^[3]^ Studies indicate that factors like maternal smoking, childhood asthma, outdoor air pollution, workplace dust and fume exposure, secondhand smoke, and biomass inhalation increase the risk of COPD, making a country like Nepal more vulnerable.^[4,5]^

The World Health Organization has identified Kathmandu, Nepal, as one of the most polluted cities in Asia, particularly because of elevated levels of particulate matter-10 (PM10) and PM2.5 in the air, with PM2.5 concentration being 7.3 times higher than the recommended air quality guideline.^[6,7]^ With prolonged exposure to pollutants because of infrastructural development, high emissions from automobiles, factories, and an increasing rate of urbanization, traffic police are exposed to automobile pollution every day, resulting in increased vulnerability to COPD.^[8]^ The age-standardized incidence rate of chronic respiratory diseases (CRDs) in Nepal was reported to be 913.6 per 100,000 population. Among the CRDs, most of the disability adjusted life years (DALY) were attributable to COPD.^[9]^ The DALY was reported to be 2274.9 per 100,000 population and 2013.3 per 100,000 population for females and males, respectively.^[10]^ Despite the substantial national burden of COPD and the high vulnerability of traffic police personnel, there is scarce information about knowledge of COPD among traffic police.^[11]^

Health literacy helps improve knowledge and understanding of health determinants and can motivate people to adopt healthy behaviors.^[12]^ It is crucial for traffic police to be well-informed about COPD to implement effective practices and interventions, reducing the risk of respiratory problems they may face. Few studies have been conducted to assess knowledge and practices for preventing respiratory health problems. To date, no research has been done to evaluate COPD literacy among traffic police. This encourages us to further examine the level of awareness and understanding of COPD among traffic police.^[4]^ This study aims to determine the knowledge of chronic obstructive pulmonary disease among traffic police in Kathmandu Valley.

## 2. Methodology

### 2.1. Design, study population

**Figure 1.**
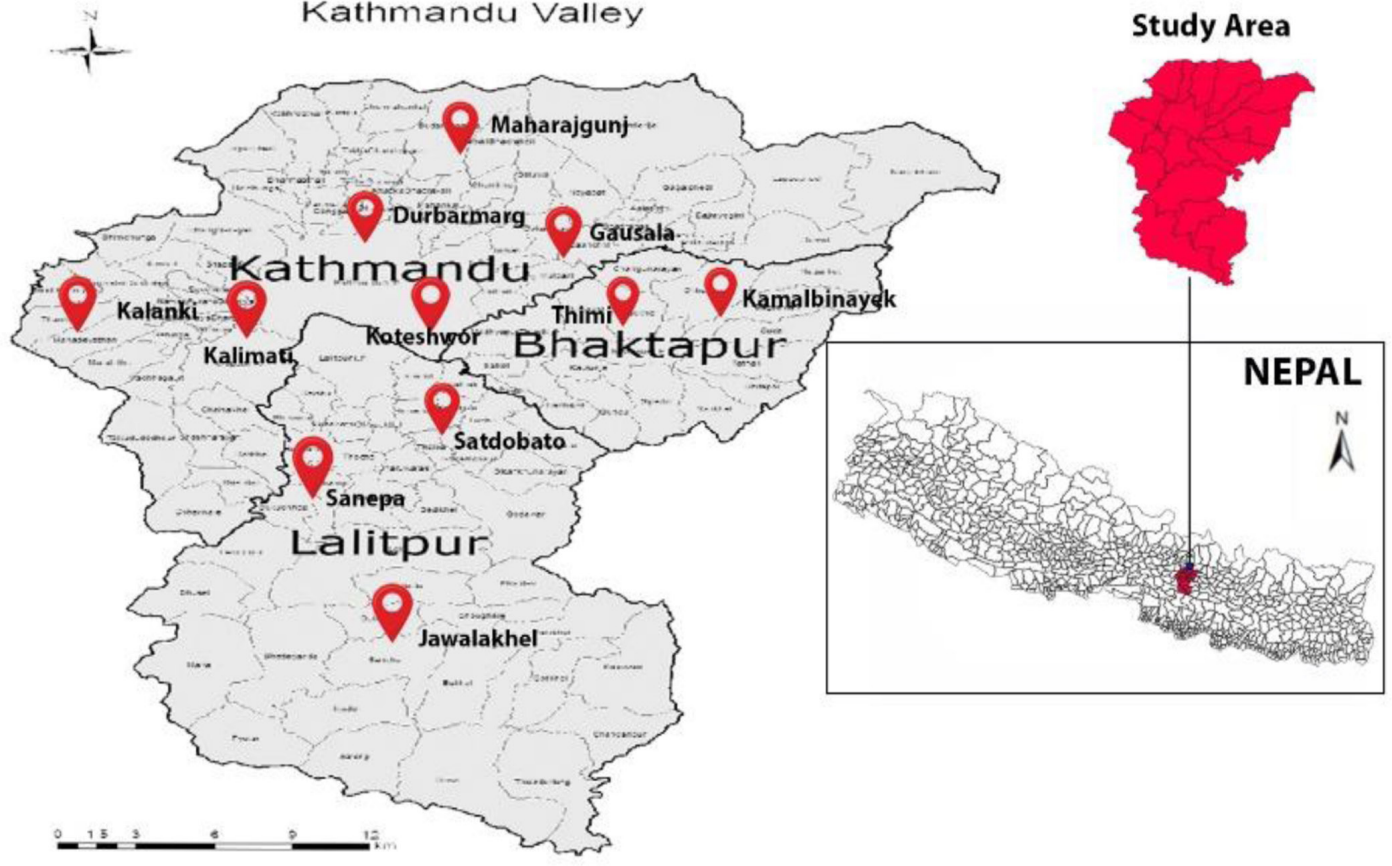
Study location for COPD literacy among traffic police in Kathmandu. COPD = chronic obstructive pulmonary disease.

This study used a descriptive cross-sectional with structured questionnaire. The study population consisted of traffic police who were working inside Kathmandu Valley (Kathmandu, Lalitpur, and Bhaktapur) at the time of the study. Out of the total 59 traffic police sites within Kathmandu Valley, 11 sites were selected through proportionate random sampling, and data were collected from July to August 2023. The study locations are marked on the map below in Figure 1.

The sampling frame included traffic police of Kathmandu Valley in various randomly selected locations. Sample size was determined using the formula^[13]^:


$$N=\frac{Z^{2}\times \pi \left(1-\pi \right)}{\delta^{2}}$$

Here, *N* is the desired sample size, *Z* is the standard normal deviate, which is set to 1.96 corresponding to a 95% confidence interval (CI). π is the estimated porportion (prevalence) in the target population with a prevalence of COPD. The reported prevalence of COPD from community studies varied widely from 1.67% to 14.3%,^[14]^ so we are taking the value of π to be 14.3%. The maximum permissible error (𝛿) was set at 5%. With the consideration of a 10% nonresponse rate, the sample size was calculated to be 208. The sample size taken was 250.

**Figure 2.**
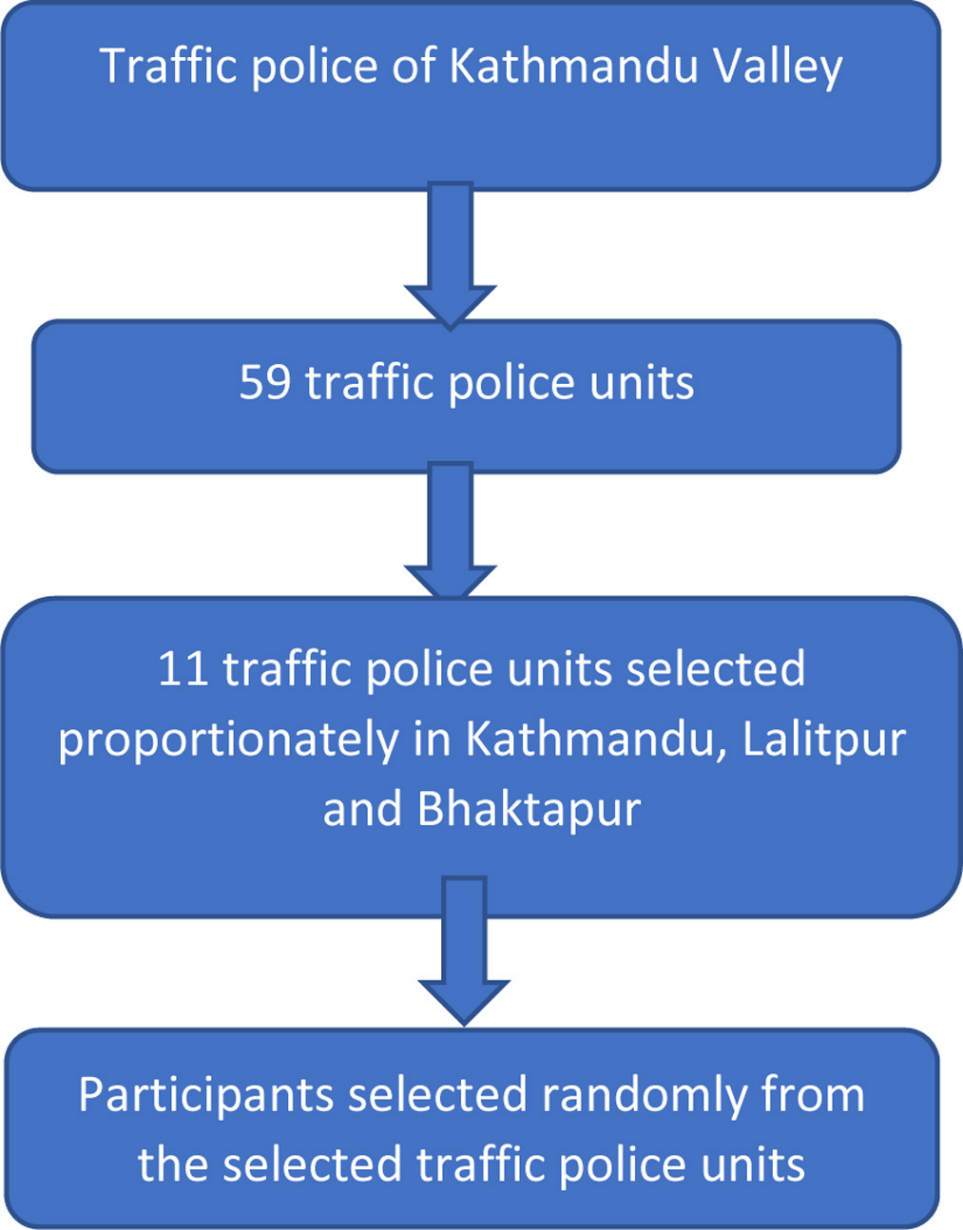
Flowchart for participants’ sampling process.

The study in Kathmandu Valley utilized the proportionate random sampling technique, detailed in Figure 1.^[15]^ A comprehensive list of all traffic police, a total of 59 units in the valley, was obtained from the traffic police headquarters in Ramshah Path, Kathmandu. District-wise analysis revealed that Kathmandu had the highest number of traffic police precincts, trailed by Lalitpur and Bhaktapur. To ensure proportional representation, study sites were randomly chosen based on the number of traffic police units in each district, resulting in the selection of 11 study sites across the 3 districts. Within each selected traffic police site, eligible participants were selected using simple random sampling from the list of traffic police personnel available at the unit. Sampling continued until the allocated sample size for each unit was reached. The flowchart of the sampling process is shown in Figure 2.

### 2.2. Tools and techniques

A 12-item lower middle-income countries Chronic Obstructive Pulmonary Disease Knowledge Questionnaire (LMIC COPD-KQ) with Cronbach’s alpha 0.75 was used for data collection.^[14]^ This tool had already been in use in similar settings and populations which helped to ensure validity of the tool. The technique used for data collection was a one-on-one interview with the study participants. Data collection was carried out in 2 steps:

1. Identification of survey anchors for the participants’ recruitment.
2. Survey administration.

In the first step, traffic police eligible for the study were identified by taking help from traffic headquarters to recruit the participants to participate in the survey, and in the second step, a 20- to 25-minute interview of randomly selected participants was done in the respective study location.

The study variables are as given in Table 1.

**Table 1:** List of study variables.

Variables	Categories of variables
Sociodemographic characteristics	
Age	20–30, 31–40, >40 yr
Sex	Male, female
Education	Secondary level, high school, bachelor’s and above
Family type	Nuclear family, joint family
Marital status	Married, unmarried
Income	<30,000; >30,000
Ethnicity	Dalit, Janajati, Madhesi, Brahmin/Chhetri, and others
Living	Alone, with others
Work-related	
Work experience	<5 yr, >5 yr
Working hour	<6 h, >6 h
Behavioral	
Smokable tobacco consumption	Currently smoking, used to smoke, and never smoked
Age when started smoking	<20 yr, >20 yr
Smokeless tobacco consumption	Current consumers, used to consume, and never consumed
Alcohol consumption	Current consumers, used to consume, and never consumed
Sleep hour	<6 h, >6 h
Regular physical activity	Yes, no
Regular health checkup	Yes, no
Others	
Health insurance	Yes, no
Covid-19 infection	Yes, no
Any health workers in the family	Yes, no
Exposure to smoke and dust in the past 12 mo	Yes, no
Heard about COPD from	Health workers, media, relative/neighbour, and others

COPD = chronic obstructive pulmonary disease.

### 2.3. Data management and analysis

The data collected were exported to IBM SPSS Statistics for Windows, Version 25.0 (IBM Corp.) for data cleaning and analysis. Scoring was done for the 12-item lower middle-income countries Chronic Obstructive Pulmonary Disease Knowledge Questionnaire. The correct answer was given 1 mark, and an answer with the wrong option or “I don’t know” was assigned with 0 mark. The total knowledge score ranged from 0 to 12. The median score was used as the cutoff point to categorize participants’ knowledge level; participants scoring at or above the median (≥8) were classified as having good knowledge, while those scoring below the median (<8) were classified as having limited knowledge. For descriptive purposes, median values (quartiles) and numbers or percentages were computed, depending on the type of data. Statistical comparisons between groups were performed using contingency tables and the chi-square statistics or the Mann–Whitney *U* test, depending on the type of data and comparison. Variables with a *P* value <.1 during the bivariate analysis were entered into the regression model. Variables that fulfilled the minimum requirements for further multivariable logistic regression were thus fitted into the final regression model. Multivariable logistic regression analysis was conducted to determine the statistically significant association between explanatory variables and outcome variables. The regression model allowed for the adjustment of multiple variables and helped in controlling potentially confounding variables. The adjusted odds ratio was calculated at a 95% CI, and a *P* value <.05 was considered statistically significant.

The inclusion and exclusion criteria for the study are given below.

#### 2.3.1. Inclusion criteria

1. Traffic police personnel who were above the age of 18 years.
2. Traffic police personnel who had been working in Kathmandu Valley for the past 6 months.

#### 2.3.2. Exclusion criteria

1. Traffic police officers with any form of mental illness diagnosed by a psychiatrist.
2. Traffic police who denied participating in the survey.

### 2.4. Ethical considerations

Ethical approval was obtained from the Institutional Review Committee, Institute of Medicine (Reference number: 451 6-11 E^2^ 079/080). Prior permission was obtained from the Metropolitan Traffic Police Division, Kathmandu, and written informed consent was collected from each participant before the interview. All collected information was kept strictly confidential and accessible only to the research team. Participation was voluntary.

## 3. Results

### 3.1. Sociodemographic characteristics of study participants

A total of 250 traffic police officers participated in the study. The mean age (±standard deviation) of the participants was 30.5 (±6.6) years. The majority of participants were male (91.2%), with 8.8% being female. Other sociodemographic characteristics are shown in Table 2.

**Table 2:** Sociodemographic characteristics of study participants.

Variables	Number (%)
Age (yr; mean ± SD)	30.5 ± 6.6
20–30	151 (60.4)
31–40	79 (31.6)
>40	20 (8.0)
Sex	
Male	228 (91.2)
Female	22 (8.8)
Education	
Secondary level	78 (31.2)
High school	129 (51.6)
Bachelor’s and above	43 (17.2)
Ethnicity	
Dalit	22 (8.8)
Janajati	56 (22.4)
Madhesi	16 (6.4)
Brahmin/Chhetri	154 (61.6)
Others	2 (0.8)
Income	
<30,000	64 (25.6)
>30,000	186 (74.4)
Family	
Nuclear family	103 (41.2)
Joint family	147 (58.8)
Marital status	
Married	179 (71.6)
Unmarried	71 (28.4)

SD = standard deviation.

### 3.2. Behavioral and work-related characteristics of study participants

As shown in Table 3, among 250 participants, current smokable tobacco use was reported to be 28.0%, and smokeless tobacco use was reported to be 17.2%. Nearly half of the participants (46.0%) had consumed alcohol in the past 12 months. A majority of the participants (97.2%) worked more than 6 hours a day. More than two-thirds of the participants (74.4%) had exposure to smoke and dust in the past 12 months.

**Table 3:** Behavioral and work-related characteristics of study participants.

Variables	Number (%)
Tobacco (smokable) use status	
Used to smoke	46 (18.4)
Never smoked	134 (53.6)
Current smokers	70 (28.0)
Age when started smoking (yr)	
<20	60 (24.0)
>20	56 (22.4)
Tobacco (smokeless) use status	
Used to smoke	38 (15.2)
Never smoked	169 (67.6)
Current smokers	43 (17.2)
Alcohol consumption status	
Used to	36 (14.4)
Never	99 (39.6)
Current	115 (46.0)
Regular BP measure	
Yes	187 (74.8)
No	63 (25.2)
Experience	
<5 yr	60 (24.0)
>5 yr	190 (76.0)
Working hour	
<6 h	7 (2.8)
>6 h	243 (97.2)
Sleep hour	
<6 h	108 (43.2)
>6 h	142 (56.8)
Physical activity	
Yes	120 (48.0)
No	130 (52.0)
Health insurance	
Yes	100 (40.0)
No	150 (60.0)
Regular health checkup	
Yes	74 (29.6)
No	176 (70.4)
Covid-19 infection	
Yes	81 (32.4)
No	169 (67.6)
Respiratory infection	
Yes	33 (13.2)
No	217 (86.8)
Income	
<30,000	64 (25.6)
>30,000	186 (74.4)
Living	
Alone	21 (8.4)
With others	229 (91.6)
Wear mask during work	
Yes	187 (74.8)
No	63 (25.2)
Mask provided free of cost	
Yes	36 (14.4)
No	214 (85.6)
Difficulty while using mask	
Yes	75 (30.0)
No	175 (70.0)
Any health worker in family members	
Yes	53 (21.2)
No	197 (78.8)
Have heard about CRD	
Yes	197 (78.8)
No	53 (21.2)
Media to heard about COPD (n = 197)	
Health worker	15 (7.6)
Media (TV/radio)	112 (56.9)
Relatives/neighbor	66 (33.5)
Others	4 (2.0)
Had COPD in the past	
Yes	6 (2.4)
No	244 (97.6)
Exposure to smoke and dust in past 12 mo	
Yes	186 (74.4)
No	64 (25.6)

BP = blood pressure, COPD = chronic obstructive pulmonary disease, CRD = chronic respiratory disease.

### 3.3. COPD knowledge level among traffic police

As shown in Table 4, among 250 participants, 128 (51.2%) had good knowledge of COPD, while 122 (48.8%) had limited knowledge of COPD.

**Table 4:** COPD knowledge level among traffic police.

COPD knowledge level	Number (%)
Limited knowledge	122 (48.8)
Good knowledge	128 (51.2)

COPD = chronic obstructive pulmonary disease.

### 3.4. Factors associated with knowledge of COPD among traffic police

Unmarried participants had lower odds of having good knowledge of COPD compared with married participants (adjusted odds ratio = 0.4, 95% CI = 0.2–0.7, *P* = .003). Participants who had heard about CRD information had 3.4 times higher odds of having good knowledge of COPD compared with people who had not heard about CRD information (95% CI = 1.7–6.6, *P* < .001). Participants who reported having studied COPD in school had 2.7 times higher odds of having good knowledge of COPD than participants who had not studied COPD (95% CI = 1.5–4.7, *P* < .001). Participants who never smoked had 1.9 times higher odds of having good knowledge of COPD than those who were currently smoking (95% CI = 1.0–3.5, *P* = .024), which is shown in Table 5.

**Table 5:** Factors associated with knowledge of COPD among traffic police.

Variables	Unadjusted odds ratio (95% CI)	*P* value	Adjusted odds ratio (95% CI)	*P* value
Marital status				
Married	0.4 (0.2–0.9)	**.021**	0.4 (0.2–0.7)	**.003**
Unmarried	Ref		Ref	
Working hour				
<6 h	0.2 (0.02–2.3)	.2	0.1 (0.01–1.2)	.08
>6 h	Ref		Ref	
Heard about CRD				
Yes	2.4 (1.1–4.9)	**.018**	3.4 (1.7–6.6)	**<.001**
No	Ref		Ref	
Ever studied COPD in school				
Yes	1.8 (1.0–3.4)	**.04**	2.7 (1.5–4.7)	**<.001**
No	Ref		Ref	
Smoking status				
Used to smoke	1.5 (0.7–3.5)	.2	1.6 (0.7–3.4)	.2
Never smoked	1.7 (0.9–3.3)	.06	1.9 (1.0–3.5)	**.024**
Currently smoking	Ref		Ref	

Bold values indicate statistical significance (*P* < .05).

CI = confidence interval, COPD = chronic obstructive pulmonary disease, CRD = chronic respiratory disease.

## 4. Discussion

Because the cutoff for low health literacy differs across studies, reported prevalence estimates vary.^[16,17]^ In our study, we established a knowledge threshold, setting the median health literacy score at 8. Alarmingly, 48.8% of the traffic police participants exhibited inadequate COPD literacy, consistent with low health literacy levels in Nepal. A study on comorbid COPD patients in the Sunsari district of Nepal reported similarly low health literacy scores, indicating a broader issue.^[18]^ Among university undergraduates, 60.8% were found to have limited health literacy.^[19]^ Knowledge and awareness are among the major barriers to health in Nepal.^[20]^ Moreover, limited health literacy is a problem in Southeast Asian countries.^[21]^ To our knowledge, this is the first study to explore COPD literacy among traffic police in Nepal.

Among participants, prior awareness of CRDs was associated with 3.4 times higher odds of possessing good COPD knowledge. This underscores the importance of health literacy, relying on accessible information from healthcare and education systems, as well as comprehensible advice from health information providers.^[22]^ Studying COPD in educational institutions showed a significant association with health literacy. This finding suggests that COPD related topics shoule be included in the curriculum to increase COPD literacy. The second Canadian health report has stated that health literacy levels are usually but not always related to the levels of education.^[23]^ In our study, the general level of education did not predict better COPD literacy. This result challenges conventional findings that associate education levels with health literacy,^[24,25]^ suggesting a need for comprehensive COPD information across educational levels.

Studying COPD in school and having heard about CRDs are both likely to increase an individual’s prior COPD knowledge. While some studies incorporate prior knowledge as part of health literacy,^[26]^ a growing number of researchers consider prior knowledge to be a moderating factor that facilitates health literacy rather than constituting health literacy on its own.^[27–30]^

The traffic police personnel who had never smoked were found to have significantly better COPD literacy than those who did. This finding is consistent with results that show that having better health literacy leads to health-promoting behaviors. Better COPD literacy increases awareness of the harmful effects of smoking, which in turn discourages smoking. A study conducted among smokers and nonsmokers showed that nonsmokers had better knowledge with regard to diseases associated with smoking.^[31]^ Conversely, traffic police who smoked had comparatively worse COPD literacy. This finding is substantiated by a nationwide survey of smokers in Korea in 2015, which demonstrated that Korean smokers had low levels of COPD awareness.^[32]^ While the studies that measured health literacy and smoking have had mixed results, the majority of the findings point towards an inverse relationship, with higher health literacy associated with reduced smoking.^[33,34]^

No significant association was found between COPD literacy and gender, consistent with prior research.^[35]^ Married participants were more likely to have better COPD literacy than unmarried participants. This finding is in line with previous research indicating that marriage induces motivational and communal changes, resulting in health-promoting behaviors.^[36]^

Our findings are consistent with a US-based systematic review, which indicated no gender association but positive correlations with education, ethnicity, and age.^[35]^ In a cross-sectional study in BPKIHS Dharan, COPD knowledge was significantly associated with education on the disease.^[12]^ Notably, our findings indicate a significant association between COPD literacy and having studied it in school, contrasting with the nonsignificant relationship with higher education levels. This observation aligns with the well-documented relationship between education and health, with health literacy proposed as a potential explanatory mechanism.^[37,38]^ It has to be noted that an individual’s ability to find, understand, and act on health-related information depends not only on personal competency but also on the provision of appropriate information by healthcare systems and providers.^[22,39]^ Therefore, COPD-related information should be included as a part of comprehensive school health education to advance personal health outcomes.^[40]^

## 5. Conclusion

These findings highlight the importance of incorporating COPD-related education into the curriculum and public health initiatives, especially targeting occupational groups such as traffic police. The study underscores the role of health literacy in promoting preventive behaviors and ultimately reducing the burden of COPD. As efforts to address COPD continue, targeted educational programs and interventions aimed at improving knowledge and awareness among high-risk populations, such as traffic police, are crucial for mitigating the impact of this significant public health concern.

## Acknowledgments

The authors would like to thank the Metropolitan Traffic Police Division, Kathmandu, for granting permission to conduct this study and for their cooperation throughout the data collection process. We are sincerely grateful to all the traffic police personnel who voluntarily participated in this study and shared their time and information. We also thank the Institutional Review Committee, Institute of Medicine, for their ethical review and approval of this study. We extend our gratitude to the Nepal Health Research Council for the undergraduate research grant that supported this work.

## Author contributions

**Conceptualization:** Sandhya Niroula, Kiran Paudel, Ashok Bhurtyal.

**Data curation:** Sandhya Niroula, Kiran Paudel, Aashish Rana, Nikita Bhatta.

**Formal analysis:** Sandhya Niroula, Kiran Paudel, Gaurab Bhattarai.

**Funding acquisition:** Sandhya Niroula.

**Methodology:** Sandhya Niroula, Kiran Paudel, Gaurab Bhattarai, Subash Wagle, Sangam Shah, Aashish Rana, Dikshya Parajuli, Rojina Dangol, Nikita Bhatta, Rhea Dhakal, Aayush Adhikari, Ashok Bhurtyal.

**Project administration:** Sandhya Niroula, Gaurab Bhattarai, Nikita Bhatta, Rhea Dhakal.

**Resources:** Sandhya Niroula.

**Software:** Sandhya Niroula.

**Supervision:** Kiran Paudel, Ashok Bhurtyal.

**Writing – original draft:** Sandhya Niroula, Kiran Paudel, Gaurab Bhattarai, Subash Wagle, Sangam Shah, Aashish Rana, Dikshya Parajuli, Rojina Dangol, Nikita Bhatta, Rhea Dhakal, Aayush Adhikari, Ashok Bhurtyal.

**Writing – review & editing:** Sandhya Niroula, Kiran Paudel, Subash Wagle, Aashish Rana, Dikshya Parajuli, Rojina Dangol, Sangam Shah, Nikita Bhatta, Rhea Dhakal, Aayush Adhikari, Ashok Bhurtyal.

## References

[1] Postma DS Reddel HK ten Hacken NHT van den Berge M. Asthma and chronic obstructive pulmonary disease: similarities and differences. Clin Chest Med. 2014;35:143–56. 24507842 10.1016/j.ccm.2013.09.010

[2] World Health Organization. Chronic obstructive pulmonary disease (COPD). Geneva: WHO; 2023. Available at: https://www.who.int/news-room/fact-sheets/detail/chronic-obstructive-pulmonary-disease-(copd). Accessed June 27, 2026.

[3] Global Burden of Disease Collaborative Network. Global Burden of Disease (GBD) 2023 country profile: Nepal. Available from: https://publichealthupdate.com/global-burden-of-disease-gbd-2023-country-profile-nepal/. Accessed June 27, 2026.

[4] Dhimal M Karki KB Sharma SK. Prevalence of selected chronic non-communicable diseases in Nepal. J Nepal Health Res Counc. 2019;17:394–401. 31735938 10.33314/jnhrc.v17i3.2327

[5] Decramer M Janssens W Miravitles M. Chronic obstructive pulmonary disease. Lancet. 2012;379:1341–51. 22314182 10.1016/S0140-6736(11)60968-9 PMC7172377

[6] Aryal Bhandari A Gautam R Bhandari S. Knowledge and practice on prevention of respiratory health problems among traffic police in Kathmandu, Nepal. Int Sch Res Notices. 2015;2015:716257. 27347543 10.1155/2015/716257 PMC4897119

[7] IQAir. Kathmandu air quality index (AQI) and Nepal air pollution. 2023. Available from: https://www.iqair.com/nepal/central-region/kathmandu. Accessed November 1, 2023.

[8] Katuwal S Pandey A Acharya DK. Awareness and practices of traffic police on prevention of respiratory problems in Kathmandu. Act Sci Med. 2022;6:168–76.

[9] Adhikari TB Paudel K Paudel R. Burden and risk factors of chronic respiratory diseases in Nepal, 1990–2019: an analysis of the global burden of diseases study. Health Sci Rep. 2023;6:e1091. 36741854 10.1002/hsr2.1091 PMC9887632

[10] Adhikari TB Neupane D Kallestrup P. Burden of COPD in Nepal. Int J Chron Obstruct Pulmon Dis. 2018;13:583–9. 29445275 10.2147/COPD.S154319 PMC5810531

[11] Bajaj N Sharma T Suneja D Jain S Kumar P. Determinants of respiratory and cardiovascular health effects in traffic policemen: a perception-based comparative analysis. J Transport Health. 2017;4:30–9.

[12] Shrestha A Singh SB Khanal VK Bhattarai S Maskey R Pokharel PK. Health literacy and knowledge of chronic diseases in Nepal. Health Lit Res Pract. 2018;2:e221–30. 31294298 10.3928/24748307-20181025-01 PMC6608901

[13] Naing L Nordin RB Abdul Rahman H Naing YT. Sample size calculation for prevalence studies using Scalex and ScalaR calculators. BMC Med Res Methodol. 2022;22:209. 35907796 10.1186/s12874-022-01694-7 PMC9338613

[14] Ekezie W Jenkins AR Hall IP. The burden of chronic respiratory diseases in adults in Nepal: a systematic review. Chron Respir Dis. 2021;18:1479973121994572. 34227410 10.1177/1479973121994572 PMC8264743

[15] ResearchGate. Figure 1b: Map of Kathmandu valley; within the valley Kathmandu. Available from: https://www.researchgate.net/figure/b-Map-of-Kathmandu-valley-within-the-valley-Kathmandu-Bhaktapur-and-Lalitpur-districts_fig2_307532682. Accessed April 15 2024

[16] Wu Y Wang L Cai Z Bao L Ai P Ai Z. Prevalence and risk factors of low health literacy: a community-based study in Shanghai, China. Int J Environ Res Public Health. 2017;14:628. 28604645 10.3390/ijerph14060628 PMC5486314

[17] Zhang D Wu S Zhang Y. Health literacy in Beijing: an assessment of adults’ knowledge and skills regarding communicable diseases. BMC Public Health. 2015;15:799. 26286549 10.1186/s12889-015-2151-1 PMC4545561

[18] Yadav UN Lloyd J Hosseinzadeh H Baral KP Bhatta N Harris MF. Levels and determinants of health literacy and patient activation among multi-morbid COPD people in rural Nepal: findings from a cross-sectional study. PLoS One. 2020;15:e0233488. 32469917 10.1371/journal.pone.0233488 PMC7259703

[19] Bhusal S Paudel R Gaihre M Paudel K Adhikari TB Pradhan PMS. Health literacy and associated factors among undergraduates: a university-based cross-sectional study in Nepal. PLOS Glob Public Health. 2021;1:e0000016. 36962072 10.1371/journal.pgph.0000016 PMC10022320

[20] Budhathoki SS Pokharel PK Good S Limbu S Bhattachan M Osborne RH. The potential of health literacy to address the health related UN sustainable development goal 3 (SDG3) in Nepal: a rapid review. BMC Health Serv Res. 2017;17:237. 28347355 10.1186/s12913-017-2183-6 PMC5369219

[21] Rajah R Hassali MAA Murugiah MK. A systematic review of the prevalence of limited health literacy in Southeast Asian countries. Public Health. 2019;167:8–15. 30544041 10.1016/j.puhe.2018.09.028

[22] Poureslami I Nimmon L Rootman I Fitzgerald MJ. Health literacy and chronic disease management: drawing from expert knowledge to set an agenda. Health Promot Int. 2017;32:743–54. 26873913 10.1093/heapro/daw003 PMC5914455

[23] Kickbusch IS. Health literacy: addressing the health and education divide. Health Promot Int. 2001;16:289–97. 11509466 10.1093/heapro/16.3.289

[24] Zhang Y Zhang F Hu P. Exploring health literacy in Medical University students of Chongqing, China: a cross-sectional study. PLoS One. 2016;11:e0152547. 27050169 10.1371/journal.pone.0152547 PMC4822854

[25] Wawrzyniak AJ Ownby RL McCoy K Waldrop-Valverde D. Health literacy: impact on the health of HIV-infected individuals. Curr HIV/AIDS Rep. 2013;10:295–304. 24222474 10.1007/s11904-013-0178-4 PMC4022478

[26] Baker DW. The meaning and the measure of health literacy. J Gen Intern Med. 2006;21:878–83. 16881951 10.1111/j.1525-1497.2006.00540.x PMC1831571

[27] Paasche-Orlow MK Wolf MS. The causal pathways linking health literacy to health outcomes. Am J Health Behav. 2007;31:S19–26. 17931132 10.5555/ajhb.2007.31.supp.S19

[28] von Wagner C Steptoe A Wolf MS Wardle J. Health literacy and health actions: a review and a framework from health psychology. Health Educ Behav. 2009;36:860–77. 18728119 10.1177/1090198108322819

[29] Berkman ND Davis TC McCormack L. Health literacy: what is it? J Health Commun. 2010;15:9–19. 10.1080/10810730.2010.499985 20845189

[30] Lee SYD Arozullah AM Cho YI. Health literacy, social support, and health: a research agenda. Soc Sci Med. 2004;58:1309–21. 14759678 10.1016/S0277-9536(03)00329-0

[31] Rawbone RG Keeling CA Jenkins A Guz A. Cigarette smoking among secondary schoolchildren in 1975. Prevalence of respiratory symptoms, knowledge of health hazards, and attitudes to smoking and health. J Epidemiol Community Health (1978). 1978;32:53–8. 262590 10.1136/jech.32.1.53 PMC1087311

[32] Mun SY Hwang YI Kim JH. Awareness of chronic obstructive pulmonary disease in current smokers: a nationwide survey. Korean J Intern Med. 2015;30:191–7. 25750560 10.3904/kjim.2015.30.2.191 PMC4351325

[33] Panahi R Ramezankhani A Tavousi M Niknami S. Health literacy and smoking. J Res Health. 2018;8:1–2.

[34] Panahi R Niknami S Ramezankhani A Tavousi M Osmani F. Is there a relationship between low health literacy and smoking? Health Educ Health Promot. 2015;3:43–52.

[35] Paasche-Orlow MK Parker RM Gazmararian JA Nielsen-Bohlman LT Rudd RR. The prevalence of limited health literacy. J Gen Intern Med. 2005;20:175–84. 15836552 10.1111/j.1525-1497.2005.40245.x PMC1490053

[36] Lewis MA McBride CM Pollak KI Puleo E Butterfield RM Emmons KM. Understanding health behavior change among couples: an interdependence and communal coping approach. Soc Sci Med. 2006;62:1369–80. 16146666 10.1016/j.socscimed.2005.08.006

[37] Zajacova A Lawrence EM. The relationship between education and health: reducing disparities through a contextual approach. Annu Rev Public Health. 2018;39:273–89. 29328865 10.1146/annurev-publhealth-031816-044628 PMC5880718

[38] van der Heide I Wang J Droomers M Spreeuwenberg P Rademakers J Uiters E. The relationship between health, education, and health literacy: results from the Dutch Adult Literacy and Life Skills Survey. J Health Commun. 2013;18:172–84. 24093354 10.1080/10810730.2013.825668 PMC3814618

[39] Coleman CA Hudson S Maine LL. Health literacy practices and educational competencies for health professionals: a consensus study. J Health Commun. 2013;18:82–102. 24093348 10.1080/10810730.2013.829538 PMC3814998

[40] Belcastro PA Ramsaroop‐Hansen H. Addressing the antinomy between health education and health literacy in advancing personal health and public health outcomes. J Sch Health. 2017;87:968–74. 29096417 10.1111/josh.12570

